# *In Vitro* Anticancer Activity of *Imperata cylindrica* Root's Extract toward Human Cervical Cancer and Identification of Potential Bioactive Compounds

**DOI:** 10.1155/2021/4259777

**Published:** 2021-10-18

**Authors:** Paul Nayim, Krishna Sudhir, Armelle T. Mbaveng, Victor Kuete, Mukherjee Sanjukta

**Affiliations:** ^1^University of Dschang, Department of Biochemistry, P.O. Box 1499 Bafoussam, Dschang, Cameroon; ^2^National Centre for Biological Sciences (NCBS), Tata Institute of Fundamental Research (TIFR), Bellary Road, Bangalore, 560065 Karnataka, India

## Abstract

*Imperata cylindrica* is traditionally used to cure several diseases including cancer, wounds, and hypertension. The present study was designed to investigate the anticancer activity of the methanolic root extract of *I. cylindrica* (IC-MeOH). The water-soluble tetrazolium-1 and colony formation assays were used to check the proliferation ability of the cells. Cell apoptosis and cell cycle were measured by flow cytometry-based fluorescence-activated cell sorting. The ultrahigh-performance liquid chromatography-high-resolution mass spectrometry (UHPLC-HRMS) analysis was used for the metabolites profiling of IC-MeOH. Based on high-mass accuracy, spectral data, and previous reports, tentative compound identifications were assigned. Our findings revealed that IC-MeOH inhibited the proliferation of HeLa and CaSki cells. The plant extract was also found to induce a concentration- and time-dependent apoptosis and cell cycle arrest in the G0/G1 phase (IC_50_ value) in CaSki cell line. Analysis of IC-MeOH permitted the identification of 10 compounds already reported for their anticancer activity, epicatechin, curcumin, (-)-yatein, caffeic acid, myricetin, jatrorrhizine, harmaline, cinnamaldehyde, dobutamine, and syringin. In conclusion, IC-MeOH is a rich source of cytotoxic metabolites that inhibits human cervical cancer proliferation *via* apoptosis and cell cycle arrest.

## 1. Introduction

Cervical cancer is the fourth most common cancer in women. In 2018, an estimate of 570000 women were diagnosed with cervical cancer worldwide and about 311000 died of the disease. Almost all cervical cancer cases (99%) are caused by high-risk human papillomaviruses (HPV), an extremely common virus transmitted through sexual contact. Although most HPV infections are cured spontaneously with no symptoms, persistent infection can cause cervical cancer in women. Cervical cancer is one of the most successfully curable forms of cancer when diagnosed [1]. The chemodrugs used to treat cervical cancer include cisplatin, carboplatin, oxaliplatin, paclitaxel, and topotecan. Nonetheless, cervical cancer cells may develop resistance to cisplatin, main chemotherapy drug used for patients suffering from the cervical cancer. This substantially compromises the efficacy of cisplatin in the treatment of advanced or recurrent cervical cancer [2]. Given the fact that cisplatin can damage the kidney (nephrotoxicity) and display other common side effects such as anaphylaxis, leukopenia, neutropenia, thrombocytopenia, anaemia, hepatotoxicity, and cardiotoxicity [3], natural bioactive compounds may offer a better solution. Drug development using natural products has been extensively explored by researchers [4], and the use of plant-derived molecules is frequent in cancer research. The plant kingdom is made up of around 250000 species [5], which are regularly exposed to stressful conditions due to biotic and abiotic factors in their living environment [6]. To survive under such difficult and stressful conditions, plants undergo some important modifications leading to the synthetic stimulation of secondary metabolites, which are known for their various pharmacological activities [7]. The *in vitro* investigations of Kuete et al. [8, 9], and Nayim et al. [10] have shown the cytotoxic effects of the methanolic root extract of *I. cylindrica* against a panel of cancer cell lines including leukemia cells (CCRF-CEM and HL-60), breast cancer cells (MDA-MB-231-bcrp Clone 23), human wild-type HCT116 (P^53+/+^) colon cancer cells, and pancreatic cancer cells Mia PaCa-2. The *in vitro* study conducted by Keshava et al. [11] had revealed the weak cytotoxic effect of the methanol leaf extract of *I. cylindrica* against the human oral squamous carcinoma cell line SCC-9, and from investigations of Kwok et al. [12], the ethyl acetate extract of *I. cylindrica* leaf showed an antiproliferative activity against colorectal cancer cells HT-29. Plants' biological activities rely on their phytochemical composition, and chromatography coupled to mass spectrometry is the most widely applied technology used for the analysis of samples in very complex matrices such as plant extracts [13]. To the best of our knowledge, no research work has highlighted the anticancer mode of action of the methanolic extract of *Imperata cylindrica* root against cervical cancer. Thus, this study aimed at evaluating the metabolic profile of IC-MeOH using an ultrahigh-performance liquid chromatography-high-resolution mass spectrometry, and its anticancer mode of action toward human cervical cancer cells.

## 2. Materials and Methods

### 2.1. Collection of Plant Material

Roots of *I. cylindrica* (Gramineae) were collected in June 2019, from the “Menoua” Division in the West Region. The collected plant was identified by Mr. Nana Victor of the National Herbarium of Cameroon (Yaounde) under the voucher number 30139/SRF-Cam.

### 2.2. Extraction of Plant Material

The dried roots *of I. cylindrica* (100 g) were ground and macerated in methanol (500 mL) at room temperature for two (02) days. During extraction, the sample was shaken repeatedly. The obtained solution was filtered using Whatman No. 1 paper. Subsequently, the solvent was recovered in a rotary evaporator (BÜCHI R-Rotavapor model R-2000) and the resulting product was dried and lyophilized (Labconco Freeze Dryer—105°C, ThermoFisher, USA). The extraction yield was 7%.

### 2.3. Ultrahigh-Performance Liquid Chromatography-High-Resolution Mass Spectrometry

The ultrahigh-performance liquid chromatography-high-resolution mass spectrometry (UHPLC-HRMS) analysis was used to assess the chemical profile of IC-MeOH [14]. Seventy-five (75) mg of IC-MeOH was weighed, and 1 mL of 100% methanol was added and vortexed well. The sample was sonicated and centrifuged at 14800 rpm at 4°C for 10 min. The obtained supernatants were spiked with reserpine (positive ion mode) and Taurocholate-D8 (negative ion mode) for reverse phase and hydrophilic interaction liquid chromatography (HILIC) analyses. Afterward, the samples were vortexed and centrifuged at 14500 rpm at 4°C for 10 min. Ten (10) *μ*L of the supernatant was injected into the UHPLC-HRMS system. The mass spectrometer employed for UHPLC-HRMS analysis was a Q-Exactive Orbitrap (ThermoFisher Scientific, San Jose, CA, USA) equipped with a heated electrospray ionisation (HESI) source. It also houses a HCD (higher-energy collision dissociation) cell for carrying out MSn experiments. The Q-Exactive Orbitrap was coupled to a Dionex UltiMate 3000 UHPLC system (ThermoFisher Scientific, San Jose, CA, USA). This system was provided with a column oven (set at 40°C), an autosampler, and a thermocontroller (set at 4°C). Separation of the IC-MeOH was done using a C18 column (150 mm × 4.6.1 mm, 5 *μ*m) (Phenomenex Luna, India Pvt. Ltd.) and HILIC column (150 mm × 4.6 mm, 5 *μ*m) (Phenomenex Luna, India Pvt. Ltd.) at 40°C. Experiments were performed with sample analysis in reverse-phase chromatography (positive and negative) and HILIC (positive and negative) modes. The MS operating conditions for all three experimental replicate analyses were as follows: spray voltage, +2500 V (-2500 V for negative mode); capillary temperature, 280°C; vaporizer temperature, 320°C; sheath gas, 30 arbitrary units (40 for negative mode); and auxiliary gas, 10 arbitrary units. Injector settings were as follows: 0-2 mins: waste, 2-45 mins: load, and 45-55 mins: waste. The UHPLC-HRMS instrumentation method was used for the HILIC phase, and the UHPLC-HRMS instrumentation method for the reverse phase is compiled in Tables S1 and S2. After mass analysis, the tentative identification of compounds was achieved based on the matching of accurate *m*/*z* with local library of authentic standards from PlantCyc (10 ppm) database, METLIN Mass Spectral Database (accurate mass and/or MS/MS library), and the literature data.

### 2.4. Human Cervical Cell Line Culture

The human cervical cancer cell lines HeLa and CaSki purchased from American Type Culture Collection (ATCC, Rockville, MD, USA) were maintained in Dulbecco's Modified Eagle Medium (Gibco, ThermoFisher, USA) supplemented with 10% Foetal Bovine Serum (Gibco, ThermoFisher, USA) and 1% penicillin and streptomycin (10 mL/L) (Gibco, ThermoFisher, USA). The nontumor human cervical cell line HCK1T (donated by the National Cancer Center Research Institute (NCCRI), Japan) was maintained in 3 : 1 (*v*/*v*) Nutrient Mixture-Dulbecco's Modified Eagle Medium supplemented with 5% FBS, 0.4 *μ*g/mL hydrocortisone, 5 *μ*g/mL insulin, 8.4 ng/mL toxin cholera, 10 ng/mL epidermal growth factor, 24 ng/mL adenine, and 5 *μ*mol/mL Y-27632. All cells were incubated in a humidified atmosphere of 5% CO_2_ at 37°C. All experiments were performed with cells in the logarithmic growth phase.

### 2.5. Cell Viability Assay

The cell viability assay of IC-MeOH against HeLa, CaSki, and HCK1T cell lines was performed using the cell proliferation reagent WST-1 (Sigma-Aldrich, Roche Diagnostics, Germany) [15]. The stable tetrazolium salt WST-1 was cleaved to a soluble formazan by a complex cellular mechanism that occurs primarily at the cell surface. This bioreduction is a largely dependent dye formed directly on the glycolytic production of NAD(P)H in viable cells. Therefore, the amount of formazan dye formed directly correlates to the number of metabolically active cells in the culture. Briefly, cells were detached by treatment with 0.5% trypsin/EDTA (Gibco, Canada) and seeded at a density of 5.10^3^ cells/mL and 6.10^3^ cells/well of a 96-well cell culture plate (Greiner, Bio-One, CELLSTAR, Germany), respectively, for HeLa and CaSki in a total volume of 100 *μ*L; cells were allowed to attach overnight. Afterward, they were immediately treated with different concentrations of crude extract ranging from 3 to 384 *μ*g/mL dissolved in 0.1% dimethyl-sulfoxide (Sigma-Aldrich) and incubated for 24, 48, and 72 h; cisplatin (Celon Laboratories, India) was used as a positive control and tested from 0.1 to 100 *μ*g/mL. After the treatment periods, 10 *μ*L of WST-1 reagent was added to each well and incubated for 1 h. Absorbance was measured at 450 nm wavelength (Spectra-Max M5 Multimode reader). Each assay was performed at least three times, and the cell viability was evaluated with respect to untreated cells. IC50 values (concentration of the tested compounds required to reduce cell density to 50%) were calculated by concentration-response curve fitting using GraphPad Prism version 8.1.0.

### 2.6. Clonogenic Assay

Colony formation assay was performed to assess the effect of IC-MeOH on CaSki cell line's clonogenic ability. The following protocol has been modified from a published version [16]. Briefly, cells were harvested by trypsinization from 70 to 80% confluent monolayer cell culture, washed with PBS, and resuspended in DMEM containing 10% FBS. Afterward, cells were seeded in 6-well plates (Greiner, Bio-One, CELLSTAR, Germany) at a density of 1000 cells/well and incubated at 37°C in a humidified incubator. After 24 h, the medium was replaced with fresh medium and cells were treated with different concentrations of the plant extract (10, 15, 20, 25, and 30 *μ*g/mL) and 0.1% DMSO vehicle for 24 h. The medium was then replaced with fresh DMEM containing 10% FBS. The cells were allowed to grow for an additional 11 days. After this period, the media were removed and cells were washed with PBS, fixed with acetic acid-methanol (1 : 7 *v*/*v*), and incubated for 5 to 10 min at room temperature (RT). Afterward, colonies of cells were stained with 0.5% crystal violet (Sigma-Aldrich) and incubated for 2 h at room temperature. Cristal violet was discarded, cells were washed in tap water and dried overnight, and plates were imaged. The colonies containing at least 50 cells were counted under a Nikon inverted microscope Eclipse TE2000-S. The data were collected from three independent experiments performed in triplicate.

### 2.7. Apoptosis Analysis

A quantitative assessment of apoptosis was performed using phycoerythrin (PE) Annexin V Apoptosis Detection Kit I (BD Biosciences, Pharmagen, USA) [17]. CaSki cells were seeded in 6-well plates at a density of 4 × 10^5^ cells/well for 24 h, then treated with 0.1% DMSO or 0.3% saline, either with IC-MeOH (1/2 IC_50_, IC_50_, and 2 IC_50_) or with cisplatin used as a positive control (1/2 IC_50_, IC_50_, and 2 IC_50_) for 24 and 48 h. After the different treatment days, cells were taken out, washed twice with PBS, trypsinized, centrifuged for pellet collection, and resuspended in cold PBS and later in 1× binding buffer (1 × 10^6^ cells/mL). Afterward, 100 *μ*L of cell resuspension solution was transferred in 1.5 mL Eppendorf; 5 *μ*L of phycoerythrin-conjugated annexin V (annexin V-PE) and 5 *μ*L of 7-Amino Actinomycin D (7-AAD) were added and followed by 15 min incubation in the dark at room temperature. The stained cells were then diluted with 1× binding buffer and immediately analyzed using a flow cytometer (Becton Dickinson FACSVerse). Data from 10.000 events were collected per data file. In four zones of the drawn quadrant, we had viable cells (Q1), cells bound to annexin V-PE only (early apoptotic cells, Q2), and cells bound both to annexin-PE and 7-AAD (late apoptotic cells, Q3).

### 2.8. Cell Cycle Analysis

The effect of the methanolic root extract of *I. cylindrica* on CaSki cell cycle was determined by flow cytometry-based fluorescence-activated cell sorting (FACS) analysis of propidium iodide- (PI-) stained cells [18]. Cells were seeded in 6-well plates at a density of 1.5 × 10^5^ cells/well and incubated overnight. After serum starvation for 24 h, they were treated with either 0.1% DMSO (negative controls) or IC-MeOH (1/2 IC_50_, IC_50_, and 2 IC_50_) for 24 and 48 h. After the different treatment days, cells were trypsinized, washed with cold phosphate-buffered saline (PBS), fixed in ice-cold 70% ethanol overnight, and redissolved in 1 mL of PBS solution supplemented with RNase and stained with PI. After 15 min of incubation at 37°C, analysis was done using flow cytometry (Becton Dickinson FACSVerse). FlowJo software was used to process the data.

### 2.9. Statistical Analysis

Each experiment was performed three times, on independent cell passages. Statistical analysis was performed using GraphPad Prism version 8.1.0. The data are plotted as the mean ± SD. Differences between the means of treated and untreated samples were evaluated using one-way analysis of variance (one-way ANOVA) followed by *post hoc* Dunnett's multiple comparison test. *p*values < 0.05 were considered to be statistically significant, and significance was marked as^∗^*p* values < 0.05, ^∗∗^*p* values < 0.01, ^∗∗∗^*p* values < 0.001, and ^∗∗∗∗^*p* < 0.0001.

## 3. Results

### 3.1. The Methanol Extract of *Imperata cylindrica* Root Cytotoxicity toward Cervical Cancer Cells

**Figure 1:**
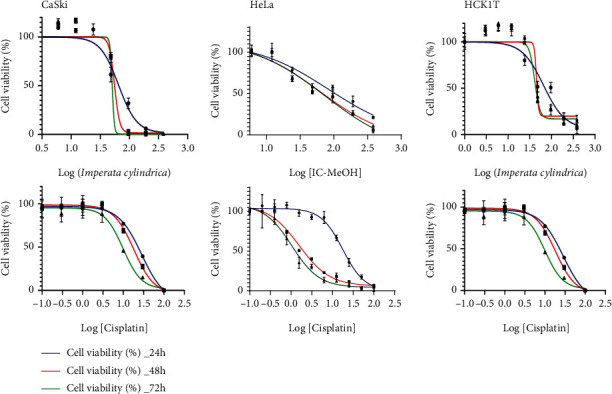
The dose-response curves of IC-MeOH and cisplatin. IC-MeOH: *Imperata cylindrica* root's methanol extract.

The antiproliferative activity of IC-MeOH on the human cervical cancer cell lines HeLa and CaSki was evaluated using the water-soluble tetrazolium-1 reagent (WST-1). Multiple concentrations of IC-MeOH and cisplatin were used, and IC_50_ values were determined from the dose-response curve. The cytotoxicity results of both IC-MeOH and the positive control against the abovementioned cell lines are shown in Figure 1 and Table 1. IC-MeOH showed a concentration- and time-dependent growth inhibition, with IC_50_ values (*μ*g/mL) of 84.17 ± 4.00, 75.05 ± 3.42, and 68.00 ± 2.39 for HeLa and 65.14 ± 3.35, 55.52 ± 0.81, and 50.51 ± 1.53 for CaSki, respectively, after 24, 48, and 72 h of treatment periods. Cisplatin also impaired HeLa and CaSki cell growth in a concentration- and time-dependent manner. However, IC-MeOH showed the best IC_50_ values with CaSki compared to HeLa. On the nontumor cervical cell line HCK1T, both IC-MeOH and cisplatin displayed a concentration- and time-dependent cytotoxicity, as their IC_50_ values on this cell line were in a decreasing order with increasing incubation periods.

### 3.2. Effect of *I. cylindrica* Root Extract on CaSki Cell Line's Clonogenic Ability

**Figure 2:**
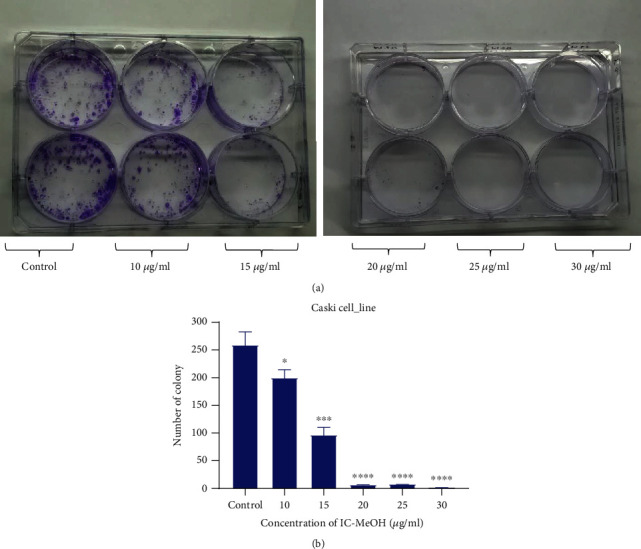
Anticlonogenic effect of IC-MeOH against CaSki cells: (a) clonogenic ability of Ca*S*ki cells in the absence (control) and in the presence of different concentrations of IC-MeOH; (b) number of colonies formed by CaSki cells in the absence (control) and in the presence of IC-MeOH and after 24 hours of treatment. All data are presented as the mean ± SD and are representative of three independent experiments. *p* values < 0.05 were considered to be statistically significant and significant, marked as ^∗^*p* values < 0.05 vs. control, ^∗∗^*p* values vs. control <0.01, ^∗∗∗^*p* values vs. control <0.001, and ^∗∗∗∗^*p* < 0.0001 vs. control. IC-MeOH: *Imperata cylindrica* root's methanol extract.

Clonogenic assay is the method of choice to determine cell reproductive death after treatment with ionising radiation but can also be used to determine the effectiveness of other cytotoxic agents. This method was used in our study to test whether IC-MeOH can reduce the clonogenic survival of CaSki cells after 24 h of treatment with different concentrations. From the obtained results displayed in Figure 2, it was notable that IC-MeOH significantly inhibited the ability of CaSki cells to form colonies at concentrations of 10, 15, 20, 25, and 30 *μ*g/mL compared to the control (untreated cells). Moreover, the anticlonogenic effect of IC-MeOH was concentration-dependent as shown by the diagram in Figure 2.

### 3.3. *I. cylindrica* Root Extract Induces Apoptosis in CaSki Cells

**Figure 3:**
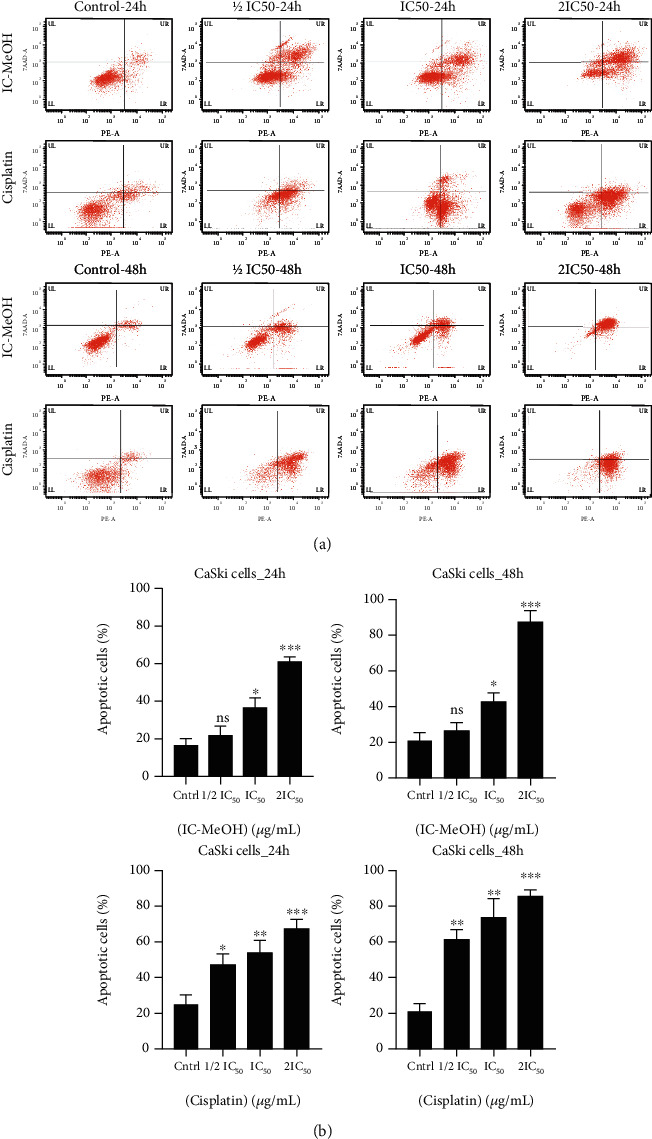
(a) Apoptotic effect of IC-MeOH in CaSki cells. Untreated CaSki cells (control) and treated CaSki cell population with IC-MeOH or cisplatin (1/2 IC_50_, IC_50_, and 2IC_50_) at different stages of apoptosis after 24 h and 48 h. Data from 10.000 cells had been collected per data file; in four zones of the drawn quadrant, we had viable cells (Q1), cells bounded to annexin V-PE only (Q2: early apoptotic cells), and cells bounded to both annexin V-PE and 7-AAD (Q3: late apoptotic cells). IC-MeOH: *Imperata cylindrica* root's methanol extract; IC_50_: inhibitory concentration 50 of IC-MeOH; ½ IC_50_: half of the inhibitory concentration 50 of IC-MeOH; 2 IC_50_: two times the inhibitory concentration 50 of IC-MeOH; 24 h, 48 h, and 72 h: treatments' times. (b) Percentage of apoptotic cells (early and late) in the control (untreated CaSki cells) and treated CaSki cells. All data presented are the mean ± SD and are representative of three independent experiments. *p* values < 0.05 were considered to be statistically significant, and significance was marked as ^∗^*p* values < 0.05, ^∗∗^*p* values < 0.01, and ^∗∗∗^*p* values < 0.001. IC-MeOH: *Imperata cylindrica* root's methanol extract; Cntrl: control; IC_50_: inhibitory concentration 50 of IC-MeOH; 1/2 IC_50_: half of the inhibitory concentration 50 of IC-MeOH; 2 IC_50_: two times the inhibitory concentration 50 of IC-MeOH; CaSki_24 h: CaSki cells treated during 24 h; CaSki_48 h: CaSki cells treated during 48 h.

7-AAD-annexin-V double staining has been used to differentiate healthy CaSki cells from early and late apoptotic CaSki cells (Figure 3(a)). Fluorescence-activated cell sorting analysis of the untreated (control) and treated cells revealed that IC-MeOH (IC_50_ and 2 IC_50_) as well as cisplatin (1/2 IC_50_, IC_50_, and 2 IC_50_) significantly induced apoptosis in CaSki cells after 24 h and 48 h of treatment. The percentages of apoptotic cells induced by IC-MeOH and cisplatin are shown in Figure 3(b).

### 3.4. *I. cylindrica* Root Extract Arrests CaSki's Cell Cycle at the G_0_/G_1_ Phase

**Figure 4:**
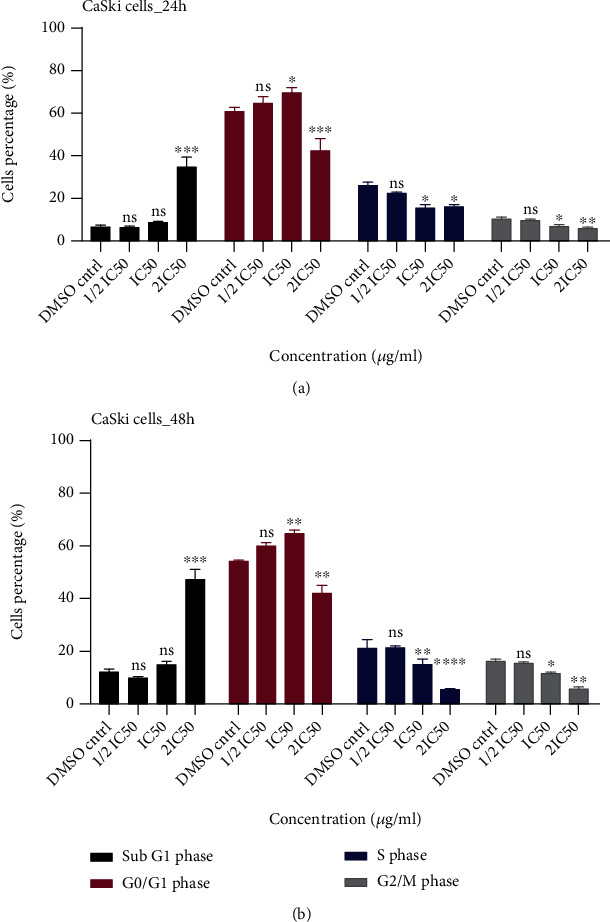
Effect of IC-MeOH on CaSki cell cycle after 24 h (a) and 48 h of treatment (b). IC-MeOH: *Imperata cylindrica* root's methanol extract; DMSO Cntrl: control (untreated cells); IC_50_: inhibitory concentration 50 of IC-MeOH; 1/2 IC_50_: half of the inhibitory concentration 50 of IC-MeOH; 2 IC_50_: two times the inhibitory concentration 50 of IC-MeOH; CaSki_24 h: CaSki cells treated during 24 h; CaSki_48 h: CaSki cells treated during 48 h.

CaSki cell cycle distribution was studied in the absence and in the presence of IC-MeOH at different concentrations equivalent to 1/2 IC_50_, IC_50_, and 2 IC_50_. Compared to untreated cells, at all treatment periods, the percentage of cells was increased at the G0/G1 phase and decreased at the S and G2/M phases in the group of cells treated at 1/2 IC_50_ and IC_50_. However, as shown in Figures 4(a) and 4(b), the aforementioned changes in cell's population percentage were significant at IC_50_ but not at 1/2 IC_50_. The same observation was made for the group cells treated at 2 IC_50_, except a significant decrease of cells at the G0/G1 phase and a large number of dead cells in the sub-G1 phase.

### 3.5. UHPLC-HRMS Analysis of *I. cylindrica* Root Methanol Extract

**Figure 5:**
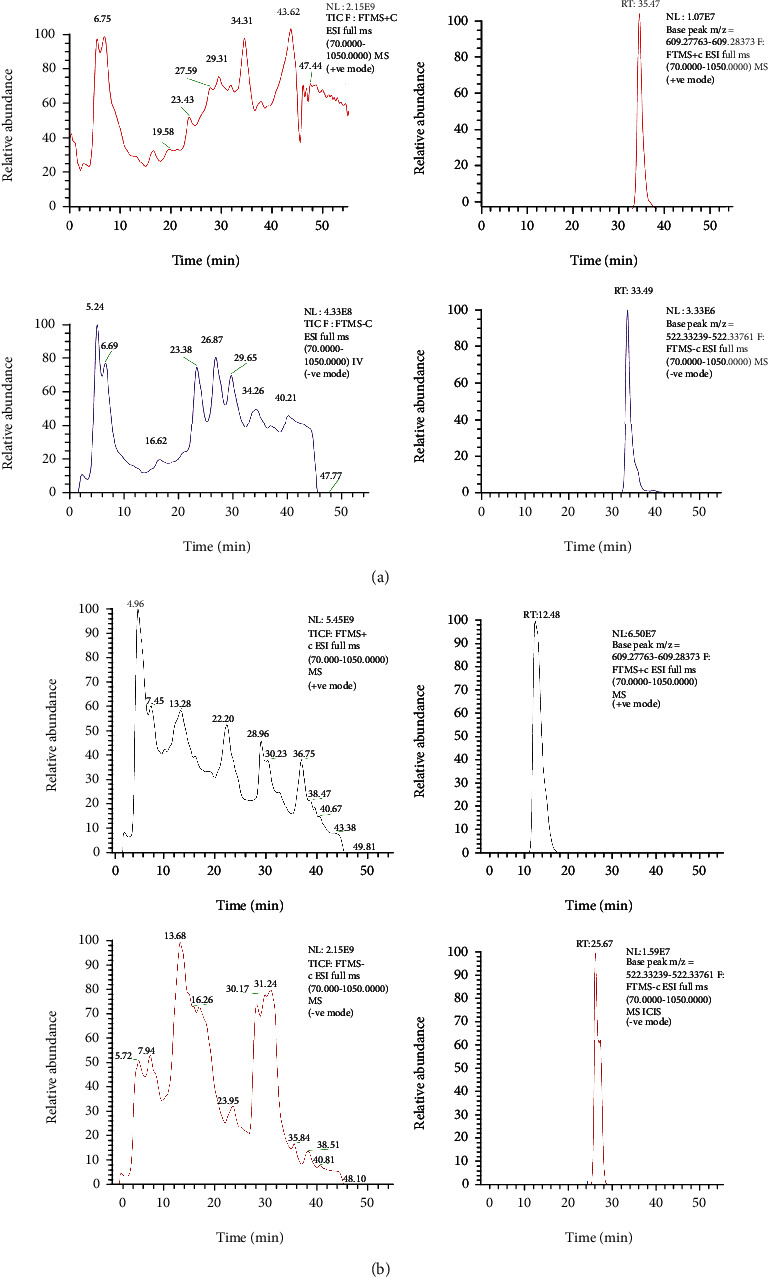
(a) UHPLC-HRMS chromatogram of IC-MeOH in the reverse phase, negative and positive ion mode. IC-MeOH: *Imperata cylindrica* root's methanol extract. (b) UHPLC-HRMS chromatogram of IC-MeOH in the HILIC phase, negative and positive ion mode. IC-MeOH: *Imperata cylindrica* root's methanol extract.

A study was conducted on the IC-MeOH based on UHPLC-HRMS in the negative and positive ion mode to identify the potential bioactive chemicals that may be responsible for its recorded anticancer activity. The chromatogram of the UHPLC-HRMS analysis of IC-MeOH is shown in Figure 5. The analysis led to the identification of 46 compounds with high-resolution mass spectrometry and MS/MS data (Table 2).

**Figure 6:**
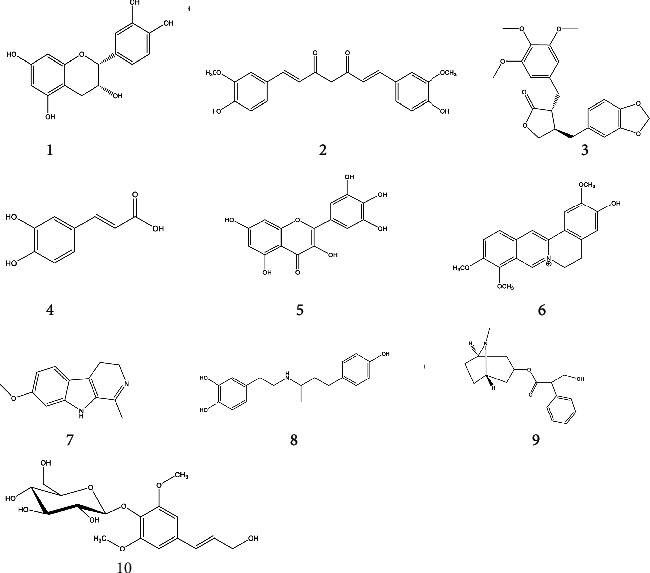
The chemical structures of some well-known identified anticancer compounds.

A total of nine compounds already reported for their anticancer activity have been tentatively identified in IC-MeOH, including 05 flavonoids (epicatechin (1), curcumin (2), (-)-yatein (3), caffeic acid (4) and myricetin (5)), 02 alkaloids (jatrorrhizine (6) and harmaline (7)), 01 phenylpropanoid (cinnamaldehyde (8)), 01 synthetic catecholamine (dobutamine (9)), and 01 monosaccharide derivative that is *trans*-sinapyl alcohol attached to a *β*-D-glucopyranosyl residue at position 1 *via* a glycosidic linkage (syringin (10)). The chemical structures of the compounds are shown in Figure 6.

## 4. Discussion

The present study was designed to identify the anticancer chemicals of IC-MeOH and to assess the inhibitory potential of the latter against human cervical cancer cell lines and the mode (s) of action (s). The UHPLC-HRMS analysis used for the metabolite profiling of IC-MeOH indicated the presence of several compounds already reported for their anticancer effects, belonging in majority to alkaloids and phenolic compounds groups. Naturally derived phenolic compounds and alkaloids are known to exhibit potent anticancer activities as well as combat various diseases through specific modes of actions. Among the identified active ingredients of IC-MeOH, epicatechin, curcumin, and myricetin are cytotoxic agents causing cancer cell death through induced apoptosis and cell cycle arrest [19–24]. Yatein is known to induce cell cycle arrest and microtubule destabilisation in human lung adenocarcinoma cells [25], and caffeic acid initiates cancer cell death by increasing intracellular ROS, altering mitochondrial membrane potential, lipid peroxidation, and apoptosis in HeLa and ME-180 cervical carcinoma cell lines [26]. Jatrorrhizine inhibits growth and induces C8161 metastatic melanoma cell cycle arrest at G0/G1 transition [27]. Harmaline induces apoptosis and prevents the proliferation and migration of human breast cancer cell lines [28]. Dobutamine displays antitumor activity against human osteosarcoma cells, *via* cell apoptosis and cell cycle arrest in the G2/M phase [29]. Cinnamaldehyde promotes apoptosis by inhibiting NF-*κ*B and AP-1 activity in cancer cells [30, 31], and syringin exhibited anticancer effects in HeLa human cervical cancer cells by inducing apoptosis, cell cycle arrest, and inhibition of cell migration [32]. The active ingredients identified in IC-MeOH are cytotoxic through apoptosis induction and cell cycle arrest. These reported findings correlate our investigations on the anticancer properties of IC-MeOH. The cytotoxicity and apoptosis assays of IC-MeOH revealed that IC-MeOH displayed a concentration- and time-dependent cytotoxicity against all the tested cell lines. Moreover, IC-MeOH inhibited the ability of CaSki cells to form colonies. The potential activity of a test substance such as a plant extract or secondary metabolite against cancer is not only associated with the cytotoxic or antiproliferative effect but is also related to the ability to inhibit mechanism concerning cancer's hallmarks [33]. The primary goal of anticancer chemotherapeutic drugs is to destroy cancer cells by inducing apoptosis in affected cells [34]. Apoptosis is a highly systematic and programmed cell death, wherein the cell debris is phagocytosed by the adjacent cells; the plant bioactive chemicals have molecular target for inducing apoptosis in different cancer cells [35–37]. In our study, IC-MeOH was found to significantly induced apoptosis in CaSki cells after 24 h and 48 h of treatment at IC_50_ and 2IC_50_ values. IC-MeOH was also found to induce G0/G1 cell cycle arrest in CaSki after 24 h and 48 h of treatment periods. These outcomes correlate with previous investigations carried out on this plant species, which revealed its *in vitro* cytotoxicity, apoptosis-induced and G0/G1 cell cycle arrest against other types of cancer, including breast cancer, blood cancer, and human liver hepatocellular carcinoma [9]. Cell cycle checkpoints can be activated by DNA damage. In this case, the growth arrest caused by checkpoints allows the cell to repair the damage. If the damage is severe and cannot be repaired, mitochondrial mechanisms kick in to convert the cell cycle arrest signal into apoptotic signal, where p53 directly and indirectly through Bax targets mitochondrial membrane potential [38]. Furthermore, cell cycle arrest in response to DNA damage activates p53 and causes a G1 arrest by inducing expression of p21 and the consequent inhibition of cyclin D/Cdk [39]. Hence, the anticancer chemicals identified in IC-MeOH may be responsible for its cytotoxicity *via* induced apoptosis and G0/G1 cell cycle arrest toward cervical cancer cells. Results displayed by Figure 1 and Table 1show higher cytotoxicity of IC-MeOH toward nontumor cells than cancer cells that could represent adverse effects. Nonetheless, our previous investigations on IC-MeOH regarding its toxicity *in vivo* revealed nontoxic effects for acute and repeated administration [40]. To the best of our knowledge, the present study is highlighting for the first time the antiproliferative mode of action of IC-MeOH toward human cervical cancer cells.

The use of herbal medicinal products for treating cancer is gaining acceptance, and many formulations have been patented and tested at the clinical trial stage.

## 5. Conclusion

The use of herbal medicinal products for treating cancer is gaining acceptance, and many formulations have been patented and tested at the clinical trial stage. The overall results provide promising baseline information to deeply investigate IC-MeOH's secondary metabolites for their anticancer activities. The UHPLC-HRMS analysis of IC-MeOH revealed the presence of anticancer chemicals belonging to various classes, which may be responsible for the cytotoxicity via apoptosis induction and G0/G1 cell cycle arrest shown by IC-MeOH toward cervical cancer cells.

## Figures and Tables

**Table 1:** IC_50_ concentrations (*μ*g/mL) of *cylindrica* root methanol extract and cisplatin.

	IC_50_ values (*μ*g/mL)								
Cell line	HeLa			CaSki			HCK1T		
Treatment time	24 h	48 h	72 h	24 h	48 h	72 h	24 h	48 h	72 h
IC-MeOH	84.17 ± 4.00	75.05 ± 3.42	68.00 ± 2.39	65.14 ± 3.35	55.52 ± 0.82	50.71 ± 1.53	65.54 ± 2.10	45.19 ± 1.70	40.91 ± 2.00
Cisplatin	17.63 ± 1.20	1.6375 ± 0.09	0.9347 ± 0.08	26.29 ± 1.70	12.07 ± 1.00	6.40 ± 0.37	28.23 ± 1.41	18.15 ± 1.01	9.83 ± 0.70

IC_50_: inhibitory concentration 50; IC-MeOH: *Imperata cylindrica* root's methanol extract.

**Table 2:** Tentative identified compounds from methanol extract of *I. cylindrica* root.

No.	MW	[M-H]^−^ (*m*/*z*)	RT (min)	MF	Tentatively identified compounds	References
1	125.014	124.0212	4.077	C_2_H_7_N0_3_S	Taurine	
2	273.966	272.9591	4.09	C_5_H_11_AsO_8_	Ribose-1-arsenate	
3	155.034	154.0424	4.885	C_3_H_10_NO_4_P	N-Methylethanolamine phosphate	
4	256.079	255.0724	4.931	C_11_H_13_NO_6_	*β*-D-Ribosylnicotinate	
5	198.052	197.0596	6.192	C_9_H_10_O_5_	Vanillylmandelic acid (100)	
6	173.069	172.0766	7.833	C_7_H1_11_NO_4_	N-Acetyl-L-glutamate 5-semialdehyde	
7	314.155	313.1624	10.193	C_20_H_18_N_4_	A bacteriochlorin	
8	153.042	152.0493	14.784	C_7_H_6_NO_3_	3-Hydroxyanthranilate	
9	225.100	224.1079	15.732	C_12_H_11_N_5_	Benzyladenine	
10	180.041	179.0345	20.723	C_9_H_8_O_4_	Caffeic acid	[41, 42]
11	339.204	338.2110	22.805	C_18_H_28_NO_5_^−^	(+)-7-Epi-12-hydroxyjasmonoyl-L-isoleucine	
12	214.110	213.1030	23.0	C_13_H_14_N_2_O	Harmaline	[41]
13	297.193	296.2012	23.445	C_16_H_27_NO_4_	N-(3-Oxododecanoyl) homoserine lactone	
14	164.057	163.0642	23.652	C_8_H_8_N_2_O_2_	Ricinine	
15	289.167	288.1746	24.843	C_17_H_23_NO_3_	Atropine	
16	346.118	345.1108	24.921	C_17_H_18_N_2_O_6_	Miraxanthin-V	
17	385.188	384.1959	25.209	C_22_H_27_NO_5_	O-Methylandrocymbine	
18	400.150	399.1572	25.220	C_22_H_24_O_7_	(-)-yatein	
19	354.165	353.1721	26.31	C_14_H_22_N_6_O_5_	Ala-His-Gln	
20	306.144	305.1513	27.242	C_13_H_18_N_6_O_3_	Lupinate	
21	380.145	379.1386	27.409	C_14_H_24_N_2_O_10_	EGTA	
22	222.088	221.0962	27.561	C_12_H_14_O_4_	Coniferyl acetate	
23	144.057	143.0642	27.797	C_10_H_8_O	1-naphthol	
24	350.174	349.1814	28.264	C_19_H_25_O_6_^−^	16, 17-Dihydro-16*α*, 17-dihydroxy gibberellin A9	
25	338.184	337.1918	28.549	C_17_H_26_N_2_O_5_	A jasmonoyl-glutamine	
26	142.063	141.0709	28.713	C_7_H_10_O_3_	Homofuraneol	
27	301.167	300.1755	29.407	C_18_H_23_NO_3_	Dobutamine	
28	318.037	317.0301	29.442	C_15_H_10_O_8_	Myricetin	
29	284.125	283.1333	29.643	C_14_H_20_O_6_	2-Phenylethyl *β*-D glucopyranoside	
30	379.246	378.2547	29.806	C_16_H_34_NO_5_P	Sphingosine 1-phosphate	
31	504.273	503.2662	30.052	C_28_H_40_O_8_	Taxusin	
32	106.041	105.0492	30.173	C_6_H_5_CHO	Benzaldehyde	
33	388.116	387.1081	30.208	C_20_H_20_O8	3,6,7,3′,4′-Pentamethylquercetagetin	
34	372.142	371.1345	30.297	C_17_H_24_O_9_	Syringin	[19]
35	132.057	131.0646	30.798	C_9_H_8_O	Cinnamaldehyde	
36	169.082	168.0896	30.898	C_9_H_11_NO_4_	L-Dihydrophenylalanine	
37	252.172	251.1791	31.638	C_15_H_24_O_3_	3-Hydroxylubimin	
38	273.193	272.2012	31.616	C_15_H_23_N	1-(p-Butylphenyl)-2,2-dimethyl-4,6-diamino-1,2-dihydro-s-Triazine	
39	338.138	337.1454	31.837	C_20_H_20_NO_4_^+1^	Jatrorrhizine	
40	440.204	399.2115	32.834	C_22_H_32_O_9_	10-Deacetyl-2-debenzoylbaccatin III	
41	518.253	517.2619	35.686	C_25_H_44_O_7_P_2_	Geranylfarnesyl diphosphate	
42	186.067	185.0755	35.887	C_12_H_10_O_2_	3,5-Dihydroxybiphenyl	
43	504.253	503.2608	36.186	C_28_H_37_FO_7_	*β*-Methasone dipropionate	
44	368.125	367.1322	37.257	C_21_H_20_O_6_	Curcumin	
45	287.151	286.1581	40.314	C_17_H_21_NO_3_	Galanthamine	
46	431.303	430.3100	41.630	C_26_H_41_NO_4_	Malyngamide H	

MW: molecular weight; RT: retention times; MF: molecular formula. Compounds were tentatively identified based on accurate *m*/*z*, standards from PlantCyc (10 ppm) database, METLIN Mass Spectral Database, and literature data.

## Data Availability

The datasets used and/or analyzed during the current study are available from the corresponding authors on reasonable request.

## Supplementary Materials

Supplementary Materials Table S1: UHPLC-HRMS instrumentation method for the HILIC phase. Table S2: UHPLC-HRMS instrumentation method for the reverse phase.

## References

[1] World Health Organization (WHO) https://www.who.int/health-topics/cervical-cancer#tab=tab_1.

[2] Zhu L., Zheng X., Du Y., Xing Y., Xu K., Cui L. (2018). Matrix metalloproteinase-7 may serve as a novel biomarker for cervical cancer. *Oncogical Targeted Therapy*.

[3] Oun R., Moussa Y. E., Yewheate N. J. (2018). The side effects of platinum-based chemotherapy drugs: a review for chemist. *Dalton Transaction*.

[4] Mbaveng A. T., Kuete V., Efferth T. (2017). Potential of Central, Eastern and Western Africa medicinal plants for cancer therapy: spotlight on resistant cells and molecular targets. *Frontiers in Pharamacology*.

[5] Narayani M., Srivastava S. (2017). Elicitation: a stimulation of stress in *in vitro* plant cell/tissue cultures for enhancement of secondary metabolite production. *Phytochemistry Reviews*.

[6] Cheynier V., Comte G., Davies K. M., Lattanzio V., Martens S. (2013). Plant phenolics: recent advances on their biosynthesis, genetics, and ecophysiology. *Plant Physiology Biochemistry*.

[7] Arnason J. T., Mata R., Romeo J. T. (2013). *Phytochemistry of Medicinal Plants*.

[8] Kuete V., Krusche B., Youns B. (2011). Cytotoxicity of some Cameroonian spices and selected medicinal plant extracts. *Journal of Ethnopharmacology*.

[9] Kuete V., Sandjo L. P., Wiench B., Efferth T. (2013). Cytotoxicity and modes of action of four Cameroonian dietary spices ethno- medically used to treat cancers: *Echinops giganteus* , *Xylopia aethiopica* , *Imperata cylindrica* and *Piper capense*. *Journal of Ethnopharmacology*.

[10] Nayim P., Mbaveng A. T., Sanjukta M., Rikesh J., Kuete V., Sudhir K. (2021). CD24 gene inhibition and TIMP-4 gene upregulation by *Imperata cylindrica*’s root extract prevents metastasis of Ca*S*ki cells *via* inhibiting PI3K/Akt/snail signaling pathway and blocking EMT. *Journal of Ethnopharmacology*.

[11] Keshava R., Muniyappa N., Rajalakshmi G., Ramaswamaiah A. S. (2016). Anti-cancer effects of *Imperata cylindrica* leaf extract on human oral squamous carcinoma cell line SCC-9 *in vitro*. *Asian Pacific Journal of Cancer Prevention*.

[12] Kwok A. H. Y., Wang Y., Ho W. S. (2016). Cytotoxic and pro-oxidative effects of *Imperata cylindrica* aerial part ethyl acetate extract in colorectal cancer *in vitro*. *Phytomedicine*.

[13] Dettmer K., Aronov P., Hammock B. (2007). Mass spectrometry-based metabolomics. *Mass Spectrometry Reviews*.

[14] Ramakrishnan P., Nair S., Rangiah K. (2016). A method for comparative metabolomics in urine using high resolution mass spectrometry. *Journal of Chromatography A*.

[15] Katarzyna B. S., Elżbieta M.-O. (2014). Biological evaluation of the activity of some benzimidazole-4, 7-dione derivatives. *Molecules*.

[16] Franken N. A., Rodermond H. M., Stap J., Haveman J., Bree V. C. (2006). Clonogenic assay of cells *in vitro*. *Natural Protocol*.

[17] Azizi M., Ghourchian H., Yazdian F., Dashtestani F., Alizadeh Zeinabad H. (2017). Cytotoxic effect of albumin coated copper nanoparticle on human breast cancer cells of MDA-MB 231. *PLoS One*.

[18] Pumiputavon K., Chaowasku T., Saenjum C. (2017). Cell cycle arrest and apoptosis induction by methanolic leaves extracts of four *Annonaceae* plants. *BMC Complementary and Alternative Medicine*.

[19] Fernando P. V., Olivares-Corichi I. M., Perez-Ruiz A. G., Luna-Arias J. O., García-Sánchez J. R. (2020). Apoptosis induced by (-)-epicatechin in human breast cancer cells is mediated by reactive oxygen species. *Molecules*.

[20] Singh M., Singh N. (2009). Molecular mechanism of curcumin induced cytotoxicity in human cervical carcinoma cells. *Molecular and Cellular Biochemistry*.

[21] Allegra A., Innao V., Russo S., Gerace D., Alonci A., Musolino C. (2017). Anticancer activity of curcumin and its analogues: preclinical and clinical studies. *Cancer Investigation*.

[22] Zang W. T., Wang Y., Wang M. (2014). Myricetin exerts anti-proliferative, anti-invasive, and pro-apoptotic effects on esophageal carcinoma EC9706 and KYSE30 cells via RSK2. *Tumor Biology*.

[23] Feng J. X., Chen Y., Wang Y., Du Q., Sun W., Zang G. Z. (2015). Myricetin inhibits proliferation and induces apoptosis and cell cycle arrest in gastric cancer cells. *Molecular and Cellular Biochemistry*.

[24] Yi J. L., Shi S., Shen Y. L. (2015). Myricetin and methyl eugenol combination enhances the anticancer activity, cell cycle arrest and apoptosis induction of cis-platin against HeLa cervical cancer cell lines. *International Journal of Clinical and Experimental Pathology*.

[25] Ho S. T., Lin C. C., Tung Y. T., Wu J. H. (2019). Molecular mechanisms underlying yatein-induced cell-cycle arrest and microtubule destabilization in human lung adenocarcinoma cells. *Cancers*.

[26] Kanimozhi G., Prasad N. R. (2015). Anticancer effect of caffeic acid on human cervical cancer cells. *Coffee in Health and Disease Prevention*.

[27] Liu R., Cao Z., Pan Y., Zhang G. (2013). Jatrorrhizine hydrochloride inhibits the proliferation and neovascularization of C8161 metastatic melanoma cells. *Anti-Cancer Drugs*.

[28] Ding Y., He J., Huang J. (2019). Harmine induces anticancer activity in breast cancer cells via targeting TAZ. *International Journal of Oncology*.

[29] Yin J., Dong Q., Zheng M. (2016). Antitumor activity of dobutamine on human osteosarcoma cells. *Oncological Letters*.

[30] Lee C. W., Lee S. H., Lee J. W. (2007). 2-Hydroxycinnamaldehyde inhibits SW620 colon cancer cell growth through AP-1 inactivation. *Journal of Pharmacological Sciences*.

[31] Cabello C. M., Warner B. B., Lamore S. D. (2009). The cinnamon-derived Michael acceptor cinnamic aldehyde impairs melanoma cell proliferation, invasiveness, and tumor growth. *Free Radical Biology and Medicine*.

[32] Xia N. (2016). Syringin exhibits anticancer effects in HeLa human cervical cancer cells by inducing apoptosis, cell cycle arrest and inhibition of cell migration. *Bangladesh Journal Pharmacology*.

[33] Hanahan D., Weinberg R. A. (2011). Hallmarks of cancer: the next generation. *Cell*.

[34] Singh R. K., Ranjan A., Srivastava A. K. (2020). Cytotoxic and apoptotic inducing activity of Amoora rohituka leaf extracts in human breast cancer cells. *Journal of Ayurveda and Integretive Medicine*.

[35] Pacifico S. M., Gallicchio P., Lorenz N. (2013). Apolar *Laurus nobilis* leaf extracts induce cytotoxicity and apoptosis towards three nervous system cell lines. *Food Chemical Toxicology*.

[36] Suresh K., Vinay S. K., Savita Y., Dey S. (2017). Antiproliferative and apoptotic effects of black turtle bean extracts on human breast cancer cell line through extrinsic and intrinsic pathway. *Chemistry Central Journal*.

[37] Majumder M., Debnath S., Gajbhiye R. L. (2019). *Ricinus communis* L. fruit extract inhibits migration/invasion, induces apoptosis in breast cancer cells and arrests tumour progression *in vivo*. *Scientific Report*.

[38] Wyllie A. H. (1997). Apoptosis: an overview. *British Medical Bulletin*.

[39] Schneider E., Montenarh M., Wagner P. (1998). Regulation of CAK kinase activity by p53. *Oncogene*.

[40] Nayim P., Mbaveng A. T., Ntyam A. M., Kuete V. (2020). A botanical from the antiproliferative Cameroonian spice, *Imperata cylindrica* is safe at lower doses, as demonstrated by oral acute and sub-chronic toxicity screenings. *BMC Complementary Medicine and Therapy*.

[41] Hyo-Jin A., Nugroho A., Song B. M., Park H. J. (2015). Isoeugenin, a novel nitric oxide synthase inhibitor isolated from the rhizomes of *Imperata cylindrica*. *Molecules*.

[42] Gebashe F., Aremu A. O., Gruz J., Finnie J. F., Staden J. V. (2020). Phytochemical profiles and antioxidant activity of grasses used in South African traditional medicine. *Plants*.

