# Supplementary material for: In Vitro Anticancer Activity of Imperata cylindrica Root's Extract toward Human Cervical Cancer and Identification of Potential Bioactive Compounds
Source: Biomed Res Int. 2021 Oct 18;2021:4259777. doi: 10.1155/2021/4259777 (PMC8545510; doi:10.1155/2021/4259777)
Supplement: Supplementary Materials — Table S1: UHPLC-HRMS instrumentation method for the HILIC phase. Table S2: UHPLC-HRMS instrumentation method for the reverse phase. [file 4259777.f1.docx]

**Supplementary material**

**S1: LC-MS Instrumentation method for HILIC phase**

***LC set-up***

| Instrument | Dionex Ultimate 3000UHPLC |
| --- | --- |
| Column | Phenomenex Luna, 5μ, HILIC,150mmx4.6mm |
| Mobile Phase A | 10mM Ammonium Acetate: Acetonitrile (1:1) (0.1%FA) |
| Mobile Phase B | Acetonitrile (0.1%FA) |
| Flow Rate | 0.4mL /min |
| Column Oven | 40^o^C |
| Auto-sampler Temp. | 4^o^C |
| Injection volume | 10μl |
| Run Time | 55 mins |
| Gradient | 0-2 mins:100%B, 2-15mins: 100-90%B, 15-25 mins: 90-80%B, 25-30 mins: 80-75%B, 30-35mins:75-20%, 35-40 mins: 20-0%B, 40-45.1: 0-100%B, 45.1-55mins: 100%B. |

***MS set-up***

| Instrument | Thermo Fisher-Q Exactive |
| --- | --- |
| Spray Voltage (+/-) | 4000/2500V |
| Vaporizer Temperature | 320^o^C |
| Sheath gas flow rate | 30 Arb |
| Aux gas flow rate | 10 Arb |
| Acquisition mode | FS, DDS (MS/MS) |
| Scan Range | 70-1050 m/z |
| Resolution | 70000, 35000 |
| Normalized Collision Energy | 35eV |
| Injector setting | 0-2mins: waste, 2-45mins: load, 45-55: waste |

**S2: LC-MS Instrumentation method for reversed phase**

***LC set-up***

| Instrument | Dionex Ultimate 3000UHPLC |
| --- | --- |
| Column | Phenomenex Luna, 5μ, C18,150mmx4.6mm |
| Mobile Phase A | 10mM Ammonium Acetate in water (0.1% FA) |
| Mobile Phase B | Acetonitrile (0.1% FA) |
| Flow Rate | 0.4mL /min |
| Column Oven | 40^o^C |
| Auto-sampler Temp. | 4^o^C |
| Injection volume | 10μl |
| Run Time | 55 mins |
| Gradient | 0-2 mins:0.2%B, 2-20 mins:0.2-20%B, 20-35mins:20-60%B, 35-40mins:60-95%B,40-45mins:95%B, 45-45,1mins: 95-0.2%B, 45.1-55mins: 0.2%B. |

***MS set-up***

| Instrument | Thermo Fisher-Q Exactive |
| --- | --- |
| Spray Voltage (+/-) | 4000/2500V |
| Vaporizer Temperature | 320^o^C |
| Sheath gas flow rate | 30 Arb |
| Aux gas flow rate | 10 Arb |
| Acquisition mode | FS, DDS (MS/MS) |
| Scan Range | 70-1050 m/z |
| Resolution | 70000, 35000 |
| Normalized Collision Energy | 35eV |
| Injector setting | 0-2mins: waste, 2-45mins: load, 45-55: waste |
